# Ringworm

**Published:** 1894-01

**Authors:** 


RINGWORM.—In his little book on Ringworm reviewed in our August number at page 446, Dr. Burnett speaks of curing ringworm with Tuberculinum. The editor of this journal tested it in one of his own cases with gratifying success, the eruption disappearing in a few days. [Editor.]

